# *EGFR* and *BRAF* mutations in inverted sinonasal papilloma — a more complex landscape?

**DOI:** 10.1007/s00428-020-02945-y

**Published:** 2020-10-13

**Authors:** Sarah Zonnur, Andreas Erbersdobler, Björn Schneider

**Affiliations:** Institute of Pathology, University Medicine Rostock, Strempelstr. 14, 18057 Rostock, Germany

**Keywords:** Genetic heterogeneity, Inverted sinonasal papilloma, Tumor evolution, *EGFR* mutation, *BRAF* mutation

## Abstract

Inverted (Schneiderian) sinonasal papilloma (ISP) is a neoplasm derived from mucosa of the sinonasal tract characterized by local aggressive growth, a tendency to recur and an association with sinonasal carcinoma. The etiology of ISP remains unclear. Recently, identical mutations in exons 19 and 20 of the oncogene *EGFR* were reported in ISP and ISP-associated sinonasal carcinoma. Nevertheless, it remains unclear whether recurring ISPs show identical *EGFR* mutations at different time points or whether these mutations are identical throughout the respective ISP sample. We used Sanger sequencing to test 60 formalin-fixed paraffin embedded ISP samples from 40 patients regarding mutations in exons 19 and 20 of *EGFR*—together with exon 15 of *BRAF*. Overall, 32 samples of 22 patients showed a mutation in *EGFR* exon 20, whereas 28 samples of 18 patients showed none. No mutation in *EGFR* exon 19 was found in any sample. Four samples of four patients showed a *BRAF* exon 15 mutation. Interestingly, samples of four patients exhibited genetic heterogeneity, enabling us to report this in ISP for the first time.

## Introduction

Inverted sinonasal papilloma (ISP) is a neoplastic proliferation arising from the sinonasal tract mucosa with predominantly inverted growth [1]. ISPs are mostly located in the nasal cavity and the maxillary sinus [2]. They often recur and may grow locally destructive [3]. The association with carcinoma is stronger for ISP than for exophytic papillomas [1]. In about 1.9 to 27% of ISPs, sinonasal squamous cell carcinomas (SSCCs) are found, the majority being synchronous tumors [4]. The etiology of ISP is virtually unknown. Exposure to organic solvents has been reported as a risk factor, and the importance of HPV-infection has been discussed, albeit controversially [2, 4].

The epidermal growth factor receptor (*EGFR*) gene is a prominent oncogene in multiple tumor entities including adenocarcinoma of the lung [5]. Here, specific mutations within exons 18–21, coding for the tyrosine kinase (TK) domain, lead to constitutive activation and thus to aberrant signaling [6]. A subset of patients with tumors showing corresponding mutations might benefit from targeted therapy with TK inhibitors [6].

Recently, *EGFR* mutations in exons 19 and 20 have been found in ISP and ISP-associated SSCC [3, 7] and their possible consequences for therapy were discussed [8]. Other driver mutations commonly found in other entities seem to be very rare [9, 10]**.**

Nevertheless, *BRAF* mutations have been shown to occur in several pre-malignant lesions like serrated adenomas of the colon [11], atypical adenomatous hyperplasia in the lung [12], endometrial hyperplasia [13], or benign melanocytic nevi of the skin [14] and the conjunctiva [15]. Therefore, we included the analysis of *BRAF* mutations in our study.

We screened for mutations in *EGFR* exon 19 and 20 and *BRAF* exon 15 in 60 FFPE samples derived of a cohort of 40 ISP patients. For 15 patients, multiple samples were available. Material from four patients showed genetic heterogeneity.

## Material and methods

### Tumor samples

Formalin-fixed paraffin embedded (FFPE) tumor material from 40 patients diagnosed with ISP was obtained from the archive of the Institute of Pathology, University Medicine, Rostock. For 15 of these patients, multiple samples were available; so altogether 60 ISP samples were analyzed. Additionally, 8 samples from 5 SSCC patients were included.

The hematoxylin and eosin (H&E) sections were examined using a BX41 light microscope (Olympus, Hamburg, Germany) to check tissue sufficiency and quality. If tumor cell ratio did not reach at least 50%, tumor tissue was dissected from slide-mounted 10μm paraffin sections before DNA extraction using a stereoscopic microscope (Technival-2, Zeiss, Jena, Germany). To check the results of each microdissection, tissue remaining on the glass slide was stained with routine H&E stains and examined microscopically.

If tumor content fell below 50% and dissection as described above was not feasible, laser capture microdissection (LCM) was performed. Briefly, 5-μm sections were mounted on Membrane Slides (MembraneSlide 1.0 PEN, Zeiss, Göttingen, Germany) and stained weakly with H&E. LCM was performed with a Zeiss Observer Z1 microscope (Zeiss) combined with a PALM Micro Beam (Zeiss) and RoboMover (Zeiss), allowing precise selection of desired cells, using PALMRobo Software V4.5 (Zeiss).

Specimen collection was conducted in accordance with the ethical guidelines for the use of human material, approved by the Ethics Committee of the University of Rostock (Reference number: A2017-0159).

### Mutation analysis

DNA was extracted from 10-μm sections of FFPE samples by deparaffinization and proteinase-K digestion (Roche, Mannheim, Germany), followed by purification with the Wizard DNA cleanup system (Promega, Mannheim, Germany) according to the manufacturer’s protocol.

For LCM obtained tissue fragments, DNA extraction was performed using the QIAamp DNA Micro Kit (Qiagen, Hilden, Germany) according to the manufacturer’s protocol for isolation of genomic DNA from laser-microdissected tissues.

DNA content was determined by fluorescent quantification (Quantus, Promega). For PCR amplification of *EGFR* exons 19 and 20 and *BRAF* exon 15, 50 ng DNA was used with the following primers: *EGFR*-19F: 5′-tgccagttaacgtcttccttctctc-3′; *EGFR*-19R: 5′-ccacacagcaaagcagaaactcac-3′; *EGFR*-20F: 5′-ccaccatgcgaagccacactga-3′ and *EGFR*-20R: 5′-tccttatctcccctccccgtatctc-3′; *BRAF*-15F: 5′-tcataatgcttgctctgatagga-3′; *BRAF*-15R: 5′-ctttctagtaactcagcagc-3′. PCR was performed using the MyTaq HS polymerase (Bioline, Luckenwalde, Germany) applying the following conditions: 95 °C for 1 min, 35 cycles of 95 °C for 15 s, 61 °C (*EGFR*) or 60 °C (*BRAF*) for 15 s, and 72 °C for 10 s. PCR products were checked by agarose gel electrophoresis. Subsequently, 15 μl thereof were purified with 3 μl Fast AP alkaline phosphatase (Thermo Scientific, Dreieich, Germany) and 1.5 μl exonuclease I (Thermo Scientific) with incubation at 37 °C for 15 min and 85 °C for 15 min.

The purified PCR products were used as template for Sanger sequencing with the abovementioned primers and BigDye™ Terminator v1.1 Cycle Sequencing Kit (Applied Biosystems, Darmstadt, Germany), substituted with BDX64 sequencing enhancing buffer (Nimagen, Nijmegen, the Netherlands). Analyses were performed on an ABI 3500 genetic analyzer (Applied Biosystems) with SeqScape software 2.7 (Applied Biosystems).

## Results

### Clinical and histological data

#### ISP patients

Sixty FFPE samples from 40 patients were analyzed. For 25 patients, only single samples were available. In all, 35 samples were derived from 15 patients (up to four samples/patient): either taken at the same time point from different (yet adjacent) locations or from the same or adjacent locations at different time points (with time intervals of up to several years) in cases of recurrent disease.

Of the 40 patients, 24 were male and 16 were female; the median age at diagnosis was 59 years (SD ± 12.7 years, range 28–86 years). Detailed clinical information is given in Table 1.

**Table 1:** Clinical data of patients and locations of obtained samples

ID no.	Gender	Age at first diagnosis (years)	Sample 1	Sample 2	Sample 3	Sample 4
			Location at first diagnosis (t1)	Location and time	Location and time	Location and time
1	Male	66	Nasal floor right	Nasal floor right (t1 + 8 years)		
2	Female	57	Nasal cavity left			
3	Male	59	Maxillary sinus left	Maxillary sinus left (t1 + 5 months)		
4	Male	43	Maxillary sinus right			
5	Male	58	Maxillary sinus right			
6	Female	70	Maxillary sinus left			
7	Female	35	Os ethmoidale post. left	Os ethmoidale left (t1)		
8	Female	55	Maxillary sinus left	Maxillary sinus left (t1 + 6 years)		
9	Male	86	Infundibulum right			
10	Male	69	Endonasal right	Nasal entrance right (t1 + 1 month)		
11	Female	69	Maxillary sinus left			
12	Female	28	Os ethmoidale left			
13	Female	66	Nasal cavity right	Processus uncinatus left, sinus left (t1 + 1 month)		
14	Male	60	Middle nasal meatus right	Nasal cavity right (t1)		
15	Male	65	Maxillary sinus right			
16	Female	75	Maxillary sinus left			
17	Male	53	Sphenoid sinus left			
18	Male	69	Os ethmoidale left	Os ethmoidale left (t1 + 7 days)		
19	Female	73	Nasal cavity left			
20	Male	44	Middle nasal meatus left	Middle nasal meatus left (t1 + 50 days)		
21	Male	53	Nasal cavity left			
22	Male	70	Os ethmoidale right			
23	Male	58	Maxillary sinus left			
24	Female	62	Sinus left			
25	Female	51	Nasal cavity right	Endonasal right (t1 + 2 years)		
26	Male	57	Maxillary sinus right			
27	Male	61	Sinus right			
28	Female	64	Nasal cavity and maxillary sinus left			
29	Male	68	Sphenoid sinus right	Sphenoid sinus right, anterior wall (t1 + 1 year 7 months)sphenoid sinus right, anterior wall (t1 + 1 year 7 months)	Sphenoid sinus right (t1 + 1 year 10 months)	
30	Male	59	Sphenoid sinus left			
31	Male	56	Nasal cavity right			
32	Male	36	Nasal cavity left	Nose right (t1 + 1 year)	Septum right (t1 + 5 years)	
33	Female	74	Nasopharynx left			
34	Female	56	Maxillary sinus left, medial wall			
35	Male	74	Concha nasalis left	Concha nasalis media left (t1 + 3 months)		
36	Male	52	Sinus frontalis right			
37	Female	51	Maxillary sinus right	Maxillary sinus right (t1 + 2 months)	Maxillary sinus right (t1 + 14 months)	Maxillary sinus right (t1 + 9 years)
38	Female	75	Os ethmoidale right			
39	Male	32	Endonasal left			
40	Female	59	Nasal entrance right	Septum right (t1)	Septum floor right (t1)	

*Abbreviations*: *t1* time point of first diagnosis

The morphology of ISP was identical in all samples and in accordance with WHO and AFIP descriptions [1, 4]. No special features could be observed in any case.

According to our current knowledge and available clinical data, none of these patients developed an ISP associated SSCC.

We observed no connection between clinical behavior (recurrence) and genetic phenotype (data given in Tables 1 and 2).

**Table 2:** Overview of mutational states in EGFR and BRAF. Abbr.: wt, wild type

ID no.	Sample 1		Sample 2		Sample 3		Sample 4	
	EGFR	BRAF	EGFR	BRAF	EGFR	BRAF	EGFR	BRAF
1	wt	wt	wt	wt				
2	wt	wt						
3	*H773dup*	wt	*H773dup*	wt				
4	*N771delinsGY*	wt						
5	*N771_P772insV*	wt						
6	wt	wt						
7	*H773dup*	wt	*H773dup*	*T589I*				
8	wt	wt	wt	wt				
9	wt	wt						
10	wt	wt	wt	wt				
11	*N771_P772insV*	wt						
12	*N771_P772insV*	wt						
13	*N771_H773dup*	wt	*N771_H773dup*	wt				
14	wt	wt	*P794L*	wt				
15	*P772L*	wt						
16	*H773_V774dup*	Q612*						
17	*D770_N771insGF*	wt						
18	wt	wt	wt	wt				
19	wt	wt						
20	wt	wt	wt	wt				
21	wt	wt						
22	wt	wt						
23	wt	wt						
24	wt	wt						
25	wt	wt	wt	wt				
26	*S768_D770dup*	wt						
27	*H773_V774dup*	wt						
28	wt	wt						
29	*H773_V774dup*	wt	wt	wt	wt	*V600E*		
30	*N771delinsGF*	wt						
31	*N771_H773dup*	wt						
32	wt	wt	wt	wt	wt	wt		
33	wt	wt						
34	*D770_N771insG*	wt						
35	*D770_P772dup*	wt	*D770_P772dup*	wt				
36	*S768_D770dup*	wt						
37	*V774_C775_insLM*	wt	*H773L, V774M*	wt	*P794L*	*H608Y*	*H773L, V774M*	wt
38	*H773dup*	wt						
39	wt	wt						
40	*S768_D770dup*	wt	*S768_D770dup*	wt	*S768_D770dup*	wt		

#### SSCC patients

For comparison, 8 samples from 5 SSCC patients were included; one diagnosed as ISP-related SSCC. One of the five SSCC patients was female, the remaining four male. The median age at diagnosis was 59 years (SD 17.4, range 48–83 years). The only patient with an ISP-associated SSCC was male, 59 years old. From this patient, three samples were available, all derived from the nasal cavity (left), at closely matching time points (after 6 or 14 days, respectively). From one the other SSCC patients, two samples were available.

### *EGFR* and *BRAF* mutation analyses

DNA extraction and Sanger sequencing of *EGFR* exons 19 and 20 and *BRAF* exon 15 were successful for all samples. No exon 19 mutation could be detected in any sample.

In 18 patients (45%), no mutations were found in any samples analyzed, while 22 patients (55%) showed a mutation. Here, for 14 patients, only single samples were analyzed and for 8 patients multiple samples. Of these 8 patients, four showed the same mutation patterns in all samples analyzed whereas another four showed genetic heterogeneity. Mutation data overview is given in Table 2.

Most *EGFR* exon 20 mutations were duplications, insertions, and other complex mutations encompassing the region around amino acid positions 768 to 774. Moreover, few point mutations within this region or at position P794 occurred. All *EGFR* exon 20 mutations lay within the TK domain. An *EGFR* exon 20 mutation occurred in at least one sample of all 22 mutation-positive patients.

*BRAF* exon 15 mutations occurred in only four patients. Only one patient (heterogeneous) showed a V600E mutation (no *EGFR* mutation in this sample), whereas the other three showed mutations near the V600 hotspot, T589I, H608Y, and Q612*, each accompanying an *EGFR* mutation.

Of the SSCC patients, the one with ISP-associated SSCC showed an *EGFR* exon 20 mutation, N771delinsGY, in all three samples. All samples of the other four SSCC patients remained wild type. No *BRAF* mutation could be detected in any SSCC sample.

### Genetic heterogeneity

**Fig. 1:**
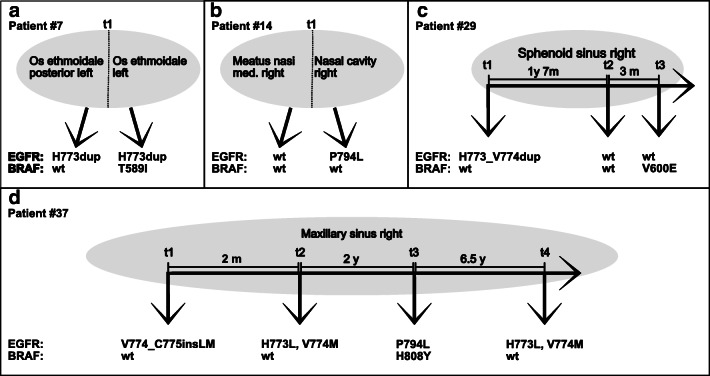
Illustration of genetic heterogeneity. **a** Patient #7 showing different mutations at the same time point at adjacent locations. **b** Patient #14 showing different mutations at the same time point at adjacent locations. **c** Patient #29 showing different mutations at the same location over time. **d** Patient #37 showing different mutations at the same location over time

Of eight mutation-positive patients with multiple samples available, four (#3, #13, #35, and #40) showed an identical mutation pattern, whereas four showed genetic heterogeneity (Fig. 1), described in detail below.

#### Patient #7

Two samples were available, taken from adjacent locations (os ethmoidale posterior left and os ethmoidale left) at the same time point. One showed an *EGFR* H773dup, the second the same *EGFR* mutation and an additional *BRAF* T589I mutation (Fig. 1a).

#### Patient #14

Two samples were taken at the same time point from adjacent locations (meatus nasalis right and nasal cavity right). The first sample was wild type for both *EGFR* and *BRAF*, while the second harbored an *EGFR* P794L mutation (Fig. 1b).

#### Patient #29

Three samples were taken over a longer time period (up to 22 months) from the same location (sphenoid sinus right). Initially, an *EGFR* H773_V774dup was observed. This mutation could not be found in the second sample taken after 1 year and 7 months: both *EGFR* and *BRAF* showed a wild-type sequence. In the third sample, taken 3 months later, a *BRAF* V600E mutation occurred while *EGFR* remained wild type (Fig. 1c).

#### Patient #37

Four samples were taken from the same location (maxillary sinus right) over a time period of almost 9 years. The tumor initially showed a complex *EGFR* V774_C775insLM mutation.

Two months later, two point mutations in *EGFR* exon 20 occurred, H773L and V774M, while no insertions were found.

At the third time point (another 2 years later), these *EGFR* codons were wild type, but a P794L point mutation had occurred. Additionally, the tumor now harbored a *BRAF* H608Y mutation. Finally, at a fourth time point (another 6.5 years later), the *EGFR* point mutations H773L and V774M re-occurred, whereas the *EGFR* P794L and *BRAF* H608Y mutations could not be detected here (Fig. 1d).

In summary, this study is the first to document the occurrence of genetic heterogeneity in ISP.

## Discussion

### *EGFR* mutations

In this study, we analyzed 60 samples taken from 40 ISP patients and found *EGFR* mutations in tissue of more than half of the patients (22/40; 55%). All *EGFR* mutations detected lay in the coding region for the TK domain of *EGFR* [6]. The only case of ISP-associated SSCC showed a N771delinsGY mutation in *EGFR* exon 20. We could not find an *EGFR* mutation in any of the four cases of non-ISP-associated SSCC investigated.

In the literature, *EGFR* mutations have been hitherto demonstrated in 38 to 88% of ISPs [3, 16] and in 50 to 88% of ISP-associated SSCCs [10, 16]: Cabal et al. found *EGFR* exon 20 mutations in 38% (7/18) of ISPs and in 50% (6/12) of ISP-associated SSCCs, while *EGFR* exon 20 mutations in ISP-unassociated SSCC occurred in only 5.3% (1/19) [16]. Sasaki et al. reported *EGFR* mutation in 100% (9/9) of ISPs and 83% (10/12) of ISPs associated with SSCC, the vast majority being exon 20 insertions. *EGFR* exon 20 mutations were also found in 88% (15/17) of ISP-associated SSCC compared with only 14% (9/63) of non-ISP-associated SSCC. The *EGFR* mutation was identical in all 12 cases of analyzed ISPs and matched ISP-associated SSCCs [10]. In the work of Udager et al., *EGFR* mutations were detected in 88% of ISPs (44/50) and in 77% (17/22) of ISP-associated SSCCs. In 83% (10/12) of cases, the identical *EGFR* mutation could be identified in ISPs and matched ISP-associated SSCCs, the other two cases being *EGFR* wild type. Most of the *EGFR* mutations reported here were *EGFR* 20 insertions [3].

While the complex *EGFR* V774_C775_insLM is has not been described hitherto, the other complex duplications have been detected in ISP [3, 17]. The point mutations H773L and V774M have yet to be described in ISP, though detected in other tumor entities, including adenocarcinoma of the lung [18, 19] and the point mutation V774M additionally in sebaceous carcinoma of the skin [20], anaplastic astrocytoma grade III [21], and phyllodes tumors of the breast [22]. The P794L and P772L mutations have also not been reported in ISP, albeit respectively discovered in cases of sebaceous adenoma [20] and adenocarcinoma of the lung [23]. The *EGFR* N771delinsGY mutation has been reported in non-small cell lung cancer [24].

### *BRAF* mutations

In four cases (10% of patients), we detected *BRAF* mutations. All detected *BRAF* mutations lay at or near the V600 hotspot. To the best of our knowledge, *BRAF* mutations have not been detected in ISP before.

Of the four samples identified with *BRAF* mutations, only one harbored a V600E mutation—well known as oncogenic driver mutation, e.g., in melanocytic tumors [25]. The three remaining samples harbored mutations at different, yet nearby loci. All these mutations have been detected but rarely in other neoplasms: H608Y in single cases of colorectal carcinoma [26, 27] and papillary thyroid carcinoma [28], T589I in a case of colon adenoma [29] and in a case of squamous cell carcinoma of the lung [30], and Q612* in a case of colon carcinoma [31] and in a case of thyroid anaplastic carcinoma [32].

### Genetic heterogeneity

In ISP samples from four patients, we found genetic heterogeneity: different genetic phenotypes could be detected in multiple samples derived of the same patient, either in samples taken at the same time from different, yet adjacent locations or derived of the same location at different time points.

Intratumoral genetic heterogeneity can develop in different ways: one implies the field cancerization theory [33] which states that different tumors can develop independently from a field of genetically altered pre-neoplastic cells [34]. This mechanism of tumorigenesis has been invoked in carcinoma of the oral cavity [34, 35] or other head and neck cancer [36]. Here, the causative agents might be tobacco smoke or alcohol and similar substances that can be inhaled or swallowed.

**Fig. 2:**
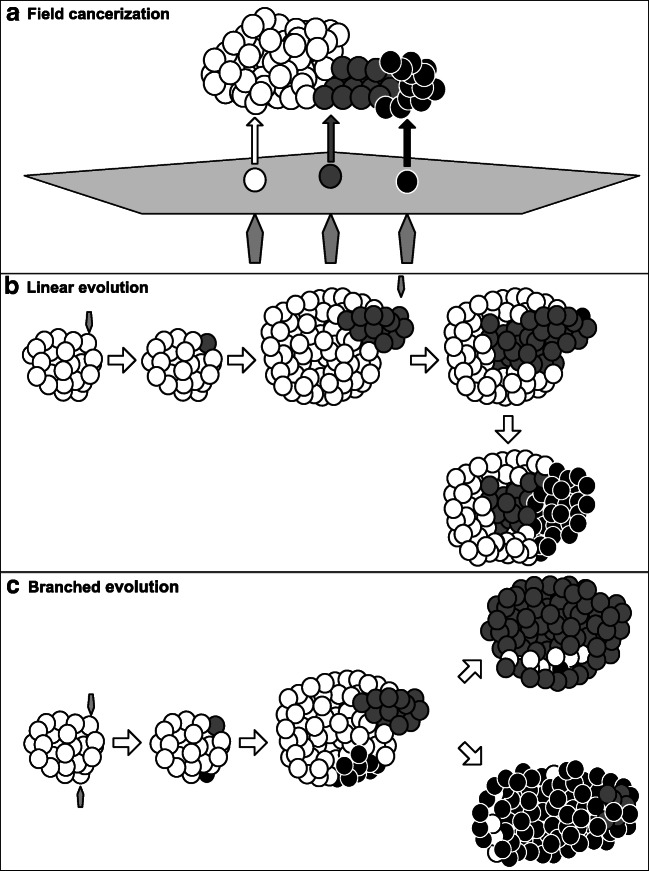
Possible causes of intratumoral genetic heterogeneity. **a** Field cancerization: exposure to noxious agents leads to several mutations (polygons) resulting in different tumor initiating cells (white, gray, and black single circles) causing a tumor mass consisting of different subclones (white, gray, and black bulks). **b** Linear evolution: in the original tumor (white) additional mutational events (polygons) leads to new subclones (at first gray, later black) which show, over time, growth advantages over the original tumor cells. **c** Branched evolution: a tumor (white) is hit by different mutational events (polygons) each leading to new subclones (gray and black), which simultaneously develop growth advantages in distinct areas

In the oral field cancerization model (field effect), the field of precancerous cells might either be derived from cells transformed by multiple events, e.g., after contact with carcinogenic substances (polyclonal field; see Fig. 2a), or by daughter cells of a single genetically altered stem cell which migrate via saliva or in the epithelium (monoclonal field) [34].

Tumorigenesis then proceeds in three phases: (1) patch formation, when the progeny of a single transformed stem cell forms a clonal proliferation; (2) clonal expansion, when the normal epithelium is replaced by genetically altered cells due to growth advantages; and (3) transition to tumor, when additional genetic alterations transform the clone into an overt carcinoma with features of invasive and metastatic potential [34]. Indeed, some have applied the theory of field cancerization not only to oral cavity, esophagus, and colon but also to skin, bladder, or vulva [37].

Clonal relationships might give important hints to the way multiple lesions have developed: if multiple lesions share common genetic alterations, they might be derived from the same progenitor cell. On the other hand, if a clonal relationship between multiple lesions is absent, they might derive from independently transformed cells [34].

Another possible cause of intratumoral heterogeneity might be tumor evolution.

In linear evolution, specific subclones may accumulate mutations which offer survival benefits. This leads to outgrowth of distinct clones and therefore temporal heterogeneity (Fig. 2b).

In branched evolution (Fig. 2c), different subclones with distinct mutation patterns exist within a tumor and may outcompete each other leading to genetic heterogeneity in samples from the same time point (reviewed in [38]).

For two of the patients showing genetic heterogeneity (#7 and #14), paired samples were taken each at the same time point from adjacent regions:

In patient #7, an *EGFR* H773dup was present in both samples, whereas in only one, a *BRAF* T589I mutation was detected. Therefore, both subclones existed in this tumor simultaneously (Fig. 1a).

In patient #14, one sample was wild type for both genes; the other harbored an *EGFR* P794L mutation. Again, both subclones existed simultaneously (Fig. 1b).

So, generally speaking, all three models of intratumoral genetic heterogeneity could apply here. However, in patient #7, due to the *EGFR* H773dup mutation shared by both subclones at the same time point, branched evolution seems more likely.

For patients #29 and #37, multiple samples were taken over longer time courses, of up to 22 months and 9 years, respectively:

Accordingly, ISP tissue in patient #29 initially showed an *EGFR* H773_V774dup mutation, which could not be detected after 19 months; so the second sample was wild type for both *EGFR* and *BRAF*. The third sample, taken 3 months later, showed a *BRAF* V600E mutation but no *EGFR* mutation (Fig. 1c).

In patient #37 (Fig. 1d), the situation was even more complex: an *EGFR* V774_C775insLM mutation was found in the first sample. Two months later, no insertions were observed, but instead, two point mutations in *EGFR* exon 20, H773L and V774M, occurred. These point mutations could not be detected at the third time point (a further 2 years later), but a P794L point mutation was present. Additionally, the tumor now harbored a de novo *BRAF* H608Y mutation. The *EGFR* point mutations H773L and V774M recurred 6.5 years later, whereas *EGFR* P794L and *BRAF* H608Y mutations could not be detected (Fig. 1d).

Tumor evolution might be a possible explanation for the loss of mutations in patients #29 and #37. In non-small cell lung cancer (NSCLC), a “loss” of *EGFR* mutation has been reported under TK inhibitor therapy. This was interpreted as a response to therapeutic pressure, where *EGFR* wild-type clones outgrew the mutant ones [39].

However, it remains unclear why ISP wild-type clones should outgrow subclones harboring a potentially activating *EGFR or BRAF* mutation.

The disappearance of these mutations might therefore raise the question whether these really offer a survival advantage for distinct subclones and, accordingly, if these mutations have clinical significance. However, since *EGFR* mutations have been reported in 38 to 88% of ISP by various groups (see above), random coincidence does not seem a likely explanation.

On the other hand, *EGFR* mutations occur in only a fraction of ISPs and ISP-associated SSCCs. So one might speculate that either another genetic alteration might be the original transforming driving event or that *EGFR* mutations and another type of driver mutation are mutually exclusive events.

Genetic heterogeneity has been increasingly identified in a variety of cancers where it may serve to promote tumor survival in response to therapy [40, 41].

The occurrence of genetic heterogeneity in benign neoplasms and precancerous lesions has received less attention. Maley et al. [42] showed that clonal diversity occurs in Barrett’s esophagus and, moreover, is linked to the risk of progression to esophageal adenocarcinoma. Others have reported subclones with different driver mutations even in small colonic adenomas [43].

Until recently, little genetic research has been performed on ISP [2]. In 2000, Califano et al. [44] investigated the random X chromosome inactivation patterns in ISP in four female patients and concluded that ISP is a monoclonal lesion. However, in one case of ISP being associated with SSCC, the inactivated X-chromosomal allele was different in the ISP and the carcinoma investigated.

Otherwise, Udager et al. [3] could demonstrate the same *EGFR* mutations in ISP and associated SSCCs in most cases and Sasaki et al. [10] in all cases analyzed.

Yakusawa et al. have investigated the genetic variants of ISP and associated SSCC in the same patients. Interestingly, they found differences: they detected one mutation in ISP alone (*GNAQ*) and two mutations in SSCC alone (*MSH6, PIK3CA*), with the majority of mutations being identical. They did not compare different samples of ISP or ISP-associated SSCC derived from the same patient [9].

The development of ISP has been linked with exposure to organic solvents and other occupational factors [45, 46], and the lesion may arise multifocally [1]. This may support the idea of a field effect in the genesis of ISP (de novo genesis of ISP)—as well as some examples of genetic heterogeneity in ISP presented here.

The question whether multiple foci of ISP or ISP-associated SSCC might share the same origin or arise independently is not purely theoretical: Udager demonstrated the sensitivity of cell lines established from ISP-associated SSCC with *EGFR* mutations to some of the new irreversible TK inhibitors [8]. Moreover, poziotinib, a third-generation EGFR inhibitor, showed initially promising results in an ongoing phase II trial of non-small cell lung cancer patients with *EGFR* exon 20 insertions [47]. In this setting, it might very well matter whether subclones with different *EGFR* mutations arise in ISP and/or in ISP-associated SSCC.

In summary, our results add to the body of knowledge regarding *EGFR* and *BRAF* mutations in ISP and show that the genetic landscape of ISP might be more complex than hitherto anticipated. Moreover, this is the first study to address testing for genetic heterogeneity in ISP samples derived from the same patient. Additionally, some of the data might be taken to support the idea of the cancer field effect in ISP. Finally, this is the first study to demonstrate *BRAF* mutations in ISP.
